# Bioactive Potential of Protein Extracts Derived from Dried *Wolffia globosa* on In Vitro Antioxidant Activities and Pro-Inflammatory Cytokine Production

**DOI:** 10.3390/molecules30204092

**Published:** 2025-10-15

**Authors:** Ruttiros Khonkarn, Krai Daowtak, Oranit Kraseasintra, Thitiya Luetragoon, Kanchana Usuwanthim, Kraisorn Taynawa, Kanokphon Chanphong

**Affiliations:** 1 Department of Pharmaceutical Science, Faculty of Pharmacy, Chiang Mai University, Chiang Mai 50200, Thailand; orkraseasintra@gmail.com; 2 Research Center of Producing and Development of Products and Innovations for Animal Health and Production, Chiang Mai University, Chiang Mai 50200, Thailand; 3 Research Center of Pharmaceutical Nanotechnology, Chiang Mai University, Chiang Mai 50200, Thailand; pei-panwad@outlook.com (K.T.); kanokphonchan@gmail.com (K.C.); 4 Department of Medical Technology, Faculty of Allied Health Sciences, Naresuan University, Phitsanulok 65000, Thailand; kraid@nu.ac.th (K.D.); thitiyal@nu.ac.th (T.L.); kanchanau@nu.ac.th (K.U.); 5 Cellular and Molecular Immunology Research Unit, Faculty of Allied Health Sciences, Naresuan University, Phitsanulok 65000, Thailand; 6 Office of Research Administration, Chiang Mai University, Chiang Mai 50200, Thailand

**Keywords:** plant-based protein, *Wolffia globosa*, nutrition, protein extraction, anti-inflammatory effects, sustainable protein

## Abstract

This study investigated the contamination, composition, and functional properties of *Wolffia globosa* from northern Thailand. The results showed that the heavy metal content of dried *W. globosa* complied with Thai regulations, ensuring its safety. Its proximate analysis revealed high protein levels with lysine, leucine, and phenylalanine as the principal essential amino acids. The protein was effectively extracted using the alkaline extraction method, followed by precipitation induced by acid or heat. The precipitates and supernatants resulting from various acid- or heat-induced protein precipitation were obtained. The highest protein content was found in the pH 3 precipitate (51.15 ± 6.71%). In contrast, the pH 5 supernatant exhibited the most potent antioxidant activities (2.22 ± 0.05 mmol Trolox/mg and 4.55 ± 0.18 mmol Fe^2+^/mg), as determined by ABTS and FRAP assays, respectively. Additionally, a strong correlation was observed between phenolic content and antioxidant activity. Both supernatant and precipitate protein extracts from *W. globosa* exhibited no cytotoxicity in THP-1 cells and displayed anti-inflammatory effects by decreasing the production of IL-1β and IL-6. They also downregulated phospho-NF-κB, phospho-IκB-α, and COX-2, consistent with reduced NF-κB pathway activation. These findings position *W. globosa* as a promising, sustainable plant-based protein with bioactive and functional properties, making it a viable candidate for functional food formulations that enhance dietary health and add value to local agricultural resources.

## 1. Introduction

As the global population is projected to exceed 9 billion by 2050, the demand for food is expected to rise by 70% [1]. This surge in demand, particularly for protein-rich foods, highlights the dual challenge of meeting nutritional requirements while ensuring environmental sustainability. Despite its role in addressing global protein needs, producing high-quality animal protein raises significant environmental concerns. Producing 1 kg of high-quality animal protein requires resources equivalent to those needed to produce approximately 6 kg of plant protein for livestock feed, placing considerable strain on land and water resources. Furthermore, this process generates substantial greenhouse gas emissions, contributing to environmental degradation and climate change [2]. In response to these challenges, novel plant-based protein sources are gaining attention for their high nutritional value and diverse bioactive components, including bioactive peptides, vitamins, minerals, and phenolic compounds. Plant-based proteins are more sustainable than animal products, as they require less land and water and generate significantly lower greenhouse gas emissions [2,3,4]. The development of such ingredients responds to the need for sustainable and accessible food innovations, in alignment with global development agendas, particularly SDG 2: Zero Hunger and SDG 3: Good Health and Well-being.

*Wolffia globosa* (*W. globosa*), known as Wolffia, Pham, or Khai nam, belongs to the Lemnaceae family. It is the smallest flowering aquatic plant, lacking a pseudo-root system, and exhibits the fastest growth rate among plants. This rootless, frond-like plant measures less than 1 mm in diameter and shows a globular or oval shape [5,6]. Native to Southeast Asia, *W. globosa* has traditionally been consumed as a vegetable in countries such as Myanmar, Laos, and northern Thailand [7]. Due to its exceptionally rapid growth rate, high protein content, and significant nutritional value, *W. globosa* has emerged as a promising candidate for sustainable food production, particularly in regions facing agricultural challenges [8,9]. Moreover, this unique plant has recently gained attention as a potential candidate for bioregenerative life support systems (BLSS), further underscoring its value in future sustainable living solutions [5].

*W. globosa* has been reported to have protein content ranging from 17% to 45%. It also contains significant amounts of chlorophyll, carotenoids, flavonoids, and essential vitamins, including cyanocobalamin (vitamin B12) [10]. This versatile nutritional profile has led to its regular use in animal feed and industrial applications, including bio alcohol production and the manufacture of biodegradable plastics [10,11]. Furthermore, *W. globosa* has garnered interest for its high levels of essential fatty acids and antioxidants, making it a promising candidate for functional foods and health-enhancing products [12,13]. In addition to its nutritional and industrial value, *W. globosa* also plays an important environmental role. It has been demonstrated to efficiently absorb nutrients, heavy metals, and organic pollutants from aquatic environments, highlighting its potential use in wastewater treatment and phytoremediation applications [9,13]. The ability to absorb contaminants further reinforces the need to evaluate heavy metal accumulation to ensure its safety as a food source.

Although *W. globosa* has attracted considerable interest as a sustainable protein source, comprehensive studies linking extraction conditions to protein yield, phenolic retention, and subsequent biofunctional activities are still limited. We hypothesized that extraction pH/temperature regulates both protein yield and phenolic retention, thereby shaping the antioxidant and anti-inflammatory properties of *W. globosa* protein extracts. Additionally, we posited that the biomass would comply with national standards for heavy metal safety. To address this hypothesis, we characterized the chemical composition of *W. globosa*, assessed heavy-metal levels against safety thresholds (Section 2.1), investigated the effect of pH/temperature on protein extraction efficiency (Section 2.2), quantified total phenolic content (Section 2.3) and antioxidant capacity (Section 2.4), and evaluated In Vitro anti-inflammatory activity (Section 2.6). By integrating nutritional analysis with mechanistic insights into the health-promoting potential of *W. globosa* protein extracts, this research provides robust evidence supporting the application of these extracts as multifunctional ingredients. These findings not only advance the understanding of agri-food bioactives but also support the development of sustainable, nutrient-rich, and therapeutically relevant functional food products.

## 2. Results

### 2.1. Chemical Compositions of Dried W. globosa (DWG)

**Figure 1:**
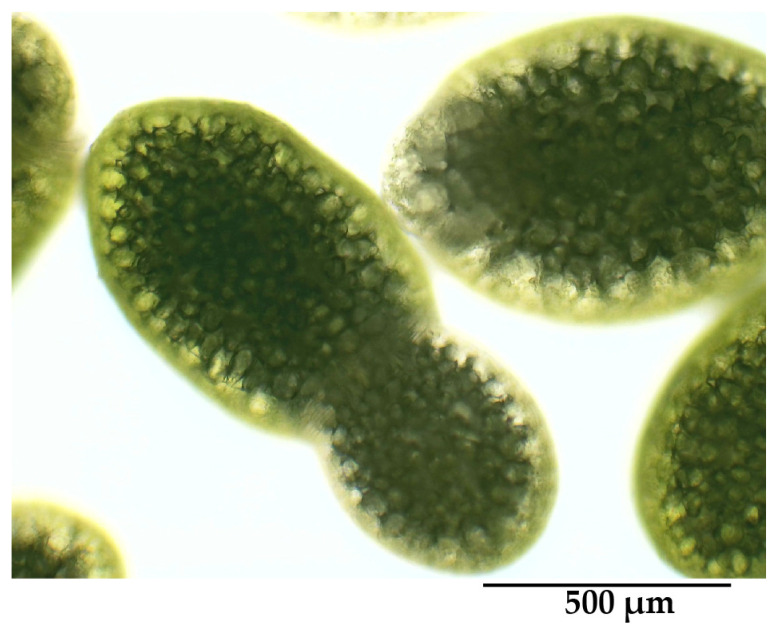
Morphological characteristics of *W. globosa* under light microscopy taken at 100× magnification. The scale bar in the lower right of the figure is 500 μm.

Wolffia samples were collected from an organic farm in San Pa Tong District, Chiang Mai, Thailand. A morphological examination identified the samples as *W. globosa*, which is characterized by elliptical fronds with light green surfaces. The frond margins exhibited a translucent appearance under top-view observation (Figure 1).

#### 2.1.1. Heavy Metal Analysis of DWG

After drying, the DWG samples were analyzed for heavy metal content. The concentrations are expressed on a dry weight basis (the moisture content of the DWG sample was 4.36% (dried weight basis). A summary of the measured values, together with the Thai regulatory limits for immediate comparison, is presented in Table 1. All values were found to be within the permissible limits.

#### 2.1.2. Proximate Analysis of DWG

The moisture content of fresh *W. globosa* was 96.51%. After drying, the DWG samples contained less than 5% moisture, confirming their stability and suitability for proximate analysis. The proximate composition analysis of DWG revealed that carbohydrates were the most abundant macronutrient, accounting for 46.16 ± 0.35 g/100 g on a dry weight basis. The protein content was also notably high at 20.23 ± 0.98 g/100 g, indicating its potential as a plant-based protein source. The fat content was relatively low, measured at 4.48 ± 0.36 g/100 g, while ash content, reflecting the mineral composition, was 15.23 ± 1.40 g/100 g.

#### 2.1.3. Amino Acid Composition

The protein quality of food materials or products is primarily determined by the type and composition of amino acids, particularly essential amino acids (EAA), while the bioactivity of peptides is influenced by both their amino acid composition and molecular mass [12,14]. In this study, the amino acid profiling of DWG revealed a total essential amino acid (EAA) content of 8608.67 mg/100 g, highlighting the presence of all nine essential amino acids (Table 2).

### 2.2. Protein Content of Supernatant (S-DWG) and Precipitate (P-DWG) Extracts of DWG

**Figure 2:**
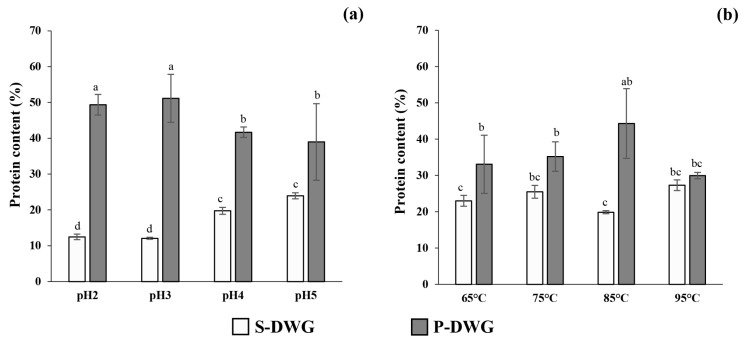
Effect of pH (**a**) and temperature (**b**) on the protein content of supernatant and precipitated extracts from DWG extracts (S-DWG and P-DWG, respectively). Results are presented as the mean ± SD from triplicate independent experiments. Different letters indicate significant differences among treatments, as determined by Duncan’s multiple range test (*p* < 0.05). The overall ANOVA *p*-value < 0.001. Detailed statistical comparisons are provided in Supplementary Tables S1 and S2.

Protein was successfully extracted from DWG using an alkaline extraction method. This method exhibited a protein recovery of 63.23% in an alkaline solution, compared to the raw material (DWG). followed by either acid or heat-induced protein precipitation (Figure 2). The protein content was consistently higher in the precipitate fractions compared to the supernatants across all conditions tested.

In the thermal precipitation series, the highest protein yield was observed at 85 °C (44.30 ± 9.62%), followed by yields at 75 °C (35.22 ± 4.00%), 65 °C (33.07 ± 8.00%), and 95 °C (29.95 ± 0.89%). Similarly, in the acid precipitation series, the highest protein content was achieved at pH 3 (51.15 ± 6.71%), followed by pH 2 (49.38 ± 2.87%), pH 4 (41.68 ± 1.46%), and pH 5 (38.97 ± 10.69%).

### 2.3. Total Phenolic Content

**Figure 3:**
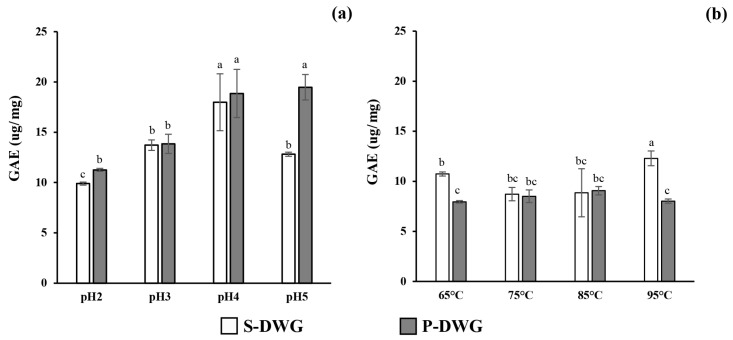
Effect of pH (**a**) and temperature (**b**) on the total phenolic content of supernatant and precipitated fractions obtained from DWG extracts (S-DWG and P-DWG, respectively). The data are expressed as mean ± SD from triplicate independent experiments. Different letters indicate significant differences among treatments, as determined by Duncan’s multiple range test (*p* < 0.05). The overall ANOVA *p*-values were < 0.001 for pH and < 0.05 for temperature. Detailed statistical results are provided in Supplementary Tables S3 and S4.

The total phenolic content (TPC) of DWG protein extracts was evaluated using the Folin–Ciocalteu assay (Figure 3). The results revealed significant differences based on extraction conditions. The highest TPC value was observed in the pH 5 precipitate (19.48 ± 1.27 µg/mg), while the lowest was found in the protein extract precipitated at 65 °C (7.95 ± 0.12 µg/mg). In general, acid-precipitated extracts demonstrated consistently higher phenolic content than those obtained Via thermal precipitation.

### 2.4. Antioxidant Activities

**Figure 4:**
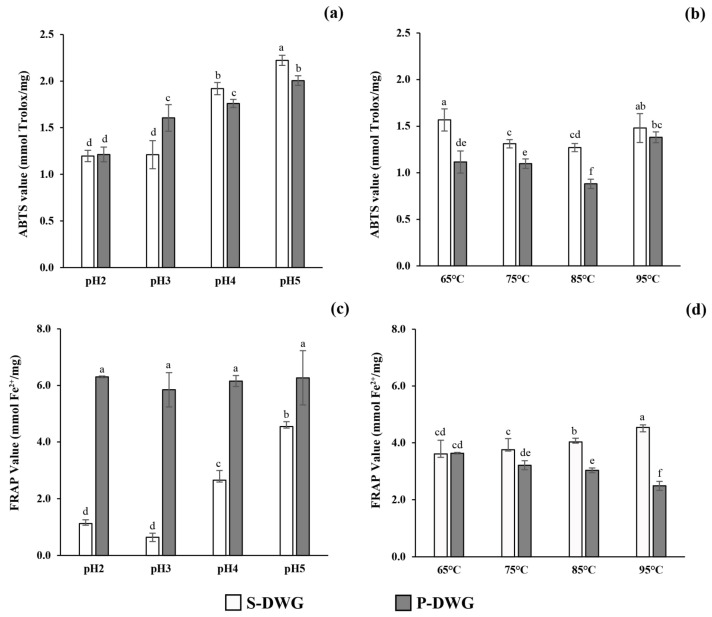
Effect of pH (**a**,**c**) and temperature (**b**,**d**) on antioxidant activities of supernatant and precipitated fractions from DWG extracts (S-DWG and P-DWG, respectively), as determined by ABTS radical scavenging (**a**,**b**) and FRAP reducing power (**c**,**d**). Results are expressed as mean ± SD from triplicate independent experiments. Different letters indicate significant differences among treatments according to Duncan’s multiple range test (*p* < 0.05).

Two antioxidant assays were employed to evaluate the free radical scavenging capacity and reducing power of DWG protein extracts. The ABTS assay was used to determine free radical scavenging activity, while the ferric ion-reducing ability was assessed using the FRAP assay. Based on the ABTS assay, the TEAC values of different DWG protein extracts ranged from 0.88 ± 0.05 to 2.22 ± 0.05 mmol Trolox/mg (Figure 4a,b). The highest TEAC value was observed in S-DWG, which was precipitated using hydrochloric acid at pH 5, while the lowest was found in P-DWG, which was heated at 85 °C. Additionally, the FRAP assay revealed EC values ranging from 0.64 ± 0.15 to 6.30 ± 0.03 mmol Fe^2+^/mg (Figure 4c,d). The highest EC value was observed in S-DWG at pH 5, whereas the lowest was found in S-DWG at pH 2. In contrast, P-DWG exhibited consistently higher FRAP values across all conditions compared to S-DWG.

### 2.5. THP-1 Cell Cytotoxicity Assay

**Figure 5:**
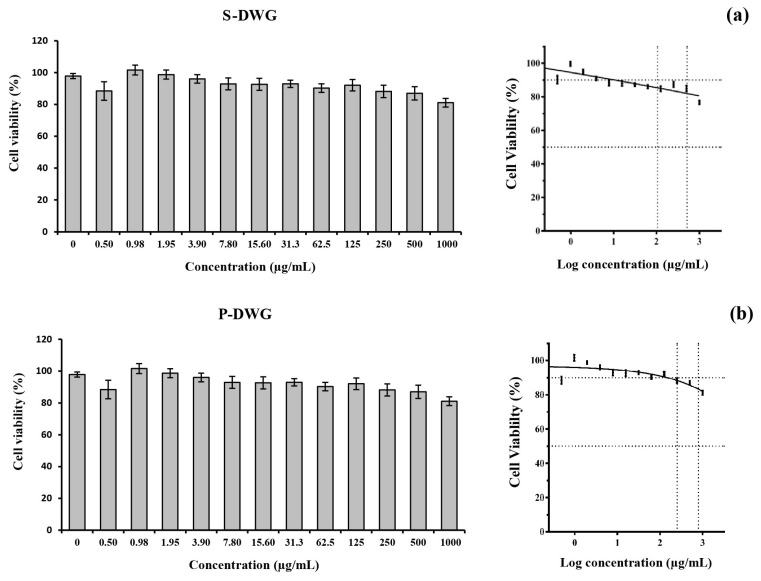
Bar graph and sigmoidal curve fitting of % cell viability for S-DWG (**a**) and P-DWG (**b**) from DWG extracts at tested concentrations of 0–1000 µg/mL.

The cytotoxic effects of DWG protein extracts, S-DWG and P-DWG, were evaluated using the MTT assay in THP-1 cells across a concentration range of 0–1000 µg/mL. As shown in Figure 5a,b, no significant reduction in cell viability was observed at any tested concentration. Both extracts-maintained cell viability above 90% across all doses. Consequently, IC_10_ and IC_50_ values could not be calculated, indicating that neither S-DWG nor P-DWG induced cytotoxic effects in the THP-1 macrophage model.

### 2.6. Effect of DWG Extracts on Pro-Inflammatory Cytokine Production

**Figure 6:**
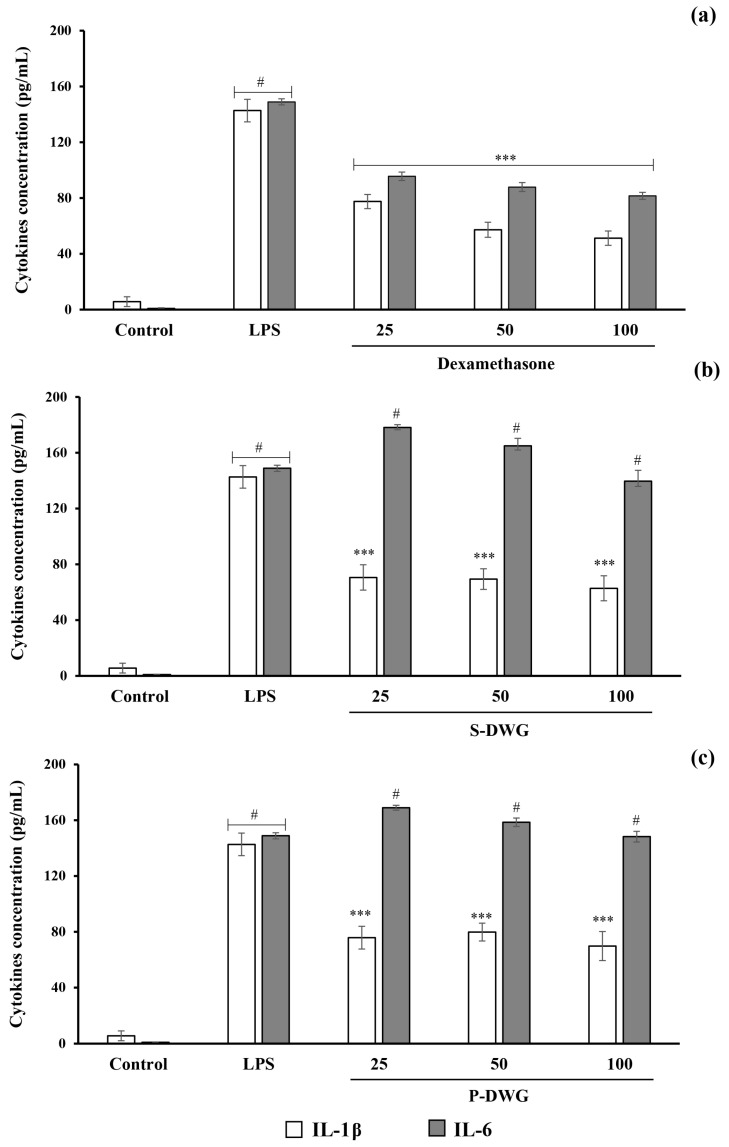
Cytokine levels in THP1-derived macrophages treated with Dexamethasone (25–100 ng/mL) (**a**), S-DWG (25–100 µg/mL) (**b**), and P-DWG (25–100 µg/mL) (**c**). The data are expressed as mean ± SEM from triplicate independent experiments. # indicates a significant difference (*p* ≤ 0.001) compared to the control; and *** (*p* ≤ 0.001) indicate significant differences compared to LPS-stimulated conditions.

The anti-inflammatory activity of DWG protein extracts was assessed by measuring the production of interleukin-1 beta (IL-1β) and interleukin-6 (IL-6) in lipopolysaccharide (LPS)-stimulated THP-1-derived macrophages. Following LPS exposure, both interleukin-1β (IL-1β) and interleukin-6 (IL-6) levels were significantly elevated, confirming activation of the pro-inflammatory response (Figure 6a). Treatment with S-DWG and P-DWG extracts significantly reduced IL-1β secretion, while IL-6 levels showed no significant change after 6 h of incubation (Figure 6b,c).

### 2.7. Effects of DWG Extracts on LPS-Induced NF-kB and COX-2 Pathway

**Figure 7:**
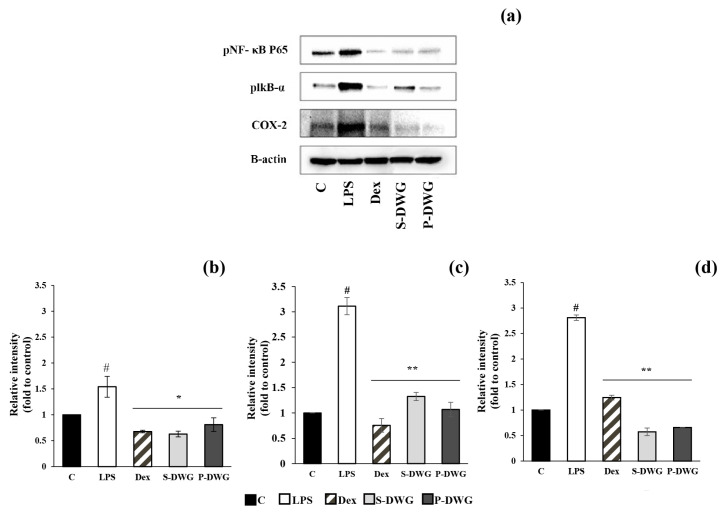
Band intensity of total protein levels of phospho-NF-kB P65, phospho-IkB-α, and COX-2 (**a**) and the bar graphs represent relative intensity of protein expression of phospho-NF-kB P65 (**b**), phospho-IkB-α (**c**), and COX-2 (**d**) from THP-1 cells tested with dexamethasone (Dex), S-DWG, and P-DWG. The data are expressed as mean ± SEM from duplicate independent experiments. # denotes a statistically significant difference (*p* ≤ 0.001) compared to the control; * (*p* ≤ 0.01), and ** (*p* ≤ 0.001) indicate significant differences compared to LPS-stimulated conditions.

The NF-κB signaling pathway, a central regulator of inflammation, was assessed to determine the anti-inflammatory effects of DWG protein extracts. THP-1-derived macrophages were stimulated with LPS to activate this pathway, followed by treatment with supernatant (S-DWG) and precipitate (P-DWG) protein extracts. As expected, LPS stimulation markedly upregulated the phosphorylation of NF-κB p65 and IκB-α and enchanced COX-2 expression compared with the control, confirming the successful induction of inflammation. Western Blot analysis showed that LPS stimulation significantly elevated the expression of phosphorylated NF-κB p65 (p-NF-κB p65), phosphorylated IκB-α (p-IκB-α), and COX-2, indicating robust activation of the inflammatory cascade (Figure 7a). Densitometric analysis confirmed that the expression of all three proteins was significantly increased compared to the control group (*p* ≤ 0.001) (Figure 7b–d).

Treatment with dexamethasone (Dex), a synthetic anti-inflammatory drug, effectively suppressed these protein expressions. Importantly, both S-DWG and P-DWG significantly reduced the expression of p-NF-κB p65 (Figure 7b), p-IκB-α (Figure 7c), and COX-2 (Figure 7d), with statistical significance ranging from *p* ≤ 0.01 to *p* ≤ 0.001 when compared to the LPS group.

## 3. Discussion

### 3.1. Chemical Compositions of DWG

The dimensions of the fronds ranged from 0.5 to 0.8 mm in length and 0.4 to 0.6 mm in width, consistent with the description of *W. globosa* as having fronds smaller than 1 mm, as reported by Crawford et al. [15]. Morphological differences based on environmental conditions were evident; plants grown in indoor reservoirs exhibited larger, dark green vesicles, whereas those from outdoor environments had smaller, yellow-green vesicles. The sample used in this study was collected in April, corresponding to the dry season in northern Thailand, when water levels are typically lower. Previous studies have reported that the growth and productivity of *W. globosa* are strongly influenced by seasonality, with peak biomass observed during the rainy season (June–October) and reduced growth during the winter and summer months, mainly due to water level fluctuations and submersion stress [16].

### 3.2. Heavy Metal Analysis of DWG

The safety of *W. globosa* for human consumption was confirmed through heavy metal analysis, with contaminant levels well below Thai regulatory limits. Exposure to heavy metals such as cadmium (Cd), lead (Pb), and arsenic (As) poses serious health risks. Cd primarily affects the kidneys and skeletal system and is a probable human carcinogen, while Pb is a neurotoxin that causes developmental impairment, particularly in children. Chronic as exposure is associated with skin lesions, cardiovascular disease, and cancers of the lung, bladder, and skin [17]. These metals are commonly introduced into aquatic environments through agricultural runoff, mining, and industrial effluents. Because *W. globosa* is a free-floating, smallest plant, it can absorb dissolved contaminants directly from the water. Recent studies have shown that *W. globosa* effectively accumulates Cd and as from polluted waters, suggesting its potential application in phytoremediation and wastewater treatment [16]. However, this same ability also highlights the importance of monitoring heavy metal levels when *W. globosa* is intended for food use. According to FAO/WHO Codex Alimentarius [18], the permissible limits for Cd and Pb in vegetables are 0.2 mg/kg and 0.3 mg/kg (fresh weight), respectively, while as should not exceed 0.5 mg/kg. In this study, all detected metal concentrations were substantially below these thresholds, confirming that the dried *W. globosa* (DWG) is safe for human consumption and suitable for use as a sustainable plant-based protein source.

### 3.3. Proximate Analysis of DWG

The chemical composition of DWG demonstrated notable nutritional value. The high carbohydrate content (46.16 ± 0.35 g/100 g DB) provides a primary energy source. In contrast, its protein content (20.23 ± 0.98 g/100 g DB) places it among notable plant-based proteins, comparable to lentils (17.5–26.2 g/100 g DB) [19] and faba beans (~28 g/100 g DB) [20]. This suggests DWG’s suitability for use in vegetarian or alternative protein diets. The fat content (4.48 ± 0.36 g/100 g DB) remains relatively low, making DWG ideal for low-fat dietary formulations. The high ash content (15.23 ± 1.4 g/100 g DB) indicates substantial mineral presence, aligning with reports of *Wolffia*’s capacity to accumulate key micronutrients [16]. Moisture content was 4.18 ± 0.49 g/100 g, confirming the sample’s stability in its dried form. When compared with other duckweed species, *W. globosa* exhibits nutrient values within or above the range reported for *Lemna minor*, which typically contains 35–43% protein and 4–6% lipid on a dry weight basis when cultivated under nutrient-rich conditions [21]. Similarly, *Spirodela polyrhiza* contains 18–28% protein and up to 5% fat, but generally lower ash content (8–10%) [16]. The relatively higher ash and balanced macronutrient profile observed in DWG highlight its nutritional robustness and suitability as a sustainable human food or functional ingredient compared to other Lemnaceae species.

### 3.4. Amino Acid Composition

DWG contained all nine essential amino acids (EAAs), with lysine (3417.67 ± 33.31 mg/100 g), leucine (1343.67 ± 41.14 mg/100 g), and phenylalanine (1360.67 ± 43.82 mg/100 g) being the most abundant. EAAs play a critical role in maintaining human health, as they are required for protein synthesis, tissue repair, enzyme function, and metabolic regulation. Specifically, lysine contributes to collagen formation and calcium absorption; leucine is a key regulator of muscle protein synthesis and energy homeostasis; and phenylalanine serves as a precursor for tyrosine and neurotransmitters such as dopamine and norepinephrine [22]. Among non-EAAs, alanine was the highest (1328.67 ± 30.66 mg/100 g), indicating support for energy metabolism. These values are consistent with previous findings [23]. The amino acid composition of DWG was compared with chicken meat, edible bird’s nest (EBN), and eggs. Compared to chicken meat, DWG has higher lysine and phenylalanine levels than chicken, with 3417.67 ± 33.31 mg/100 g and 1360.67 ± 43.82 mg/100 g in DWG, respectively, versus 1845 ± 55 mg/100 g and 800 ± 40 mg/100 g in chicken, respectively. Its histidine content (559.33 mg/100 g) is comparable to that of chicken (~610 mg/100 g). However, DWG has lower levels of methionine and glutamic acid compared to chicken, with 96 mg/100 g and 753 mg/100 g in DWG, respectively, versus ~350–500 mg/100 g and 1648 mg/100 g in chicken [24]. Additionally, DWG’s alanine and serine levels are similar to those in EBN and eggs, supporting its balanced amino acid profile [25].

Differences in amino acid composition were observed when compared to other *W. globosa* samples. Some samples had a higher total EAA content (10,743.08 mg/100 g), particularly leucine (2367.97 mg/100 g), lysine (1672.86 mg/100 g), and valine (1681.18 mg/100 g) [26], indicating that cultivation, processing, or genetic variations may affect amino acid profiles. In contrast, another *W. globosa* sample had a lower EAA proportion (~6.66 g/100 g DW) than our DWG sample [2], highlighting the importance of standardizing cultivation conditions to maximize protein content.

The complete set of essential amino acids in *W. globosa* makes it a potential candidate for alternative protein sources. Combined with its rapid growth cycle, which allows for the completion of an entire harvest within 7–14 days, it is a unique and sustainable source of protein. The following research phase aimed to optimize protein extraction methods and determine the biological activity to further enhance the functional properties of the protein.

### 3.5. Protein Content of Supernatant and Precipitate Extracts of DWG

Protein precipitation efficiency was significantly affected by both pH and temperature, with acidic conditions showing the highest efficiency (Figure 2). The highest protein yield (51.15 ± 6.71%) was obtained at pH 3, indicating that aggregation and sedimentation were favored under strongly acidic conditions (*p* < 0.001). This trend is consistent with previous findings in legume and aquatic plant proteins, where reduced electrostatic repulsion at low pH promotes protein aggregation and precipitation [14]. In contrast, the decreased protein recovery at pH 5 may be attributed to partial solubilization or the maintenance of a more native structure, leading to reduced precipitation. The balance of net charges at lower pH values facilitates intermolecular attraction between protein molecules, thereby enhancing precipitation [27].

Temperature also exerted a significant influence on protein recovery (*p* < 0.05). The optimal condition was observed at 85 °C, where moderate thermal energy likely induced partial unfolding of polypeptide chains, exposing hydrophobic residues that interact with adjacent molecules to form aggregates [28]. However, excessive heating (95 °C) appeared to cause irreversible denaturation or aggregation, thereby impairing sedimentation efficiency and resulting in lower yields.

Although direct electrophoretic or proteomic profiling was not performed in this study, previous investigations of duckweed and *Wolffia globosa* have described the predominant protein composition, which supports our observations. Nieuwland et al. [29] reported that duckweed protein concentrate contains a substantial enrichment in RuBisCO (from 48% to 92%) with an overall yield of ~27% in the concentrate. Boonarsa et al. [2] further identified prominent protein bands in *W. globosa* at approximately 62–67 kDa, potentially corresponding to structural or metabolic proteins, which accounted for approximately 38% of the total proteins. Moreover, recent optimization studies on duckweed extraction have established RuBisCO-based nitrogen-to-protein conversion factors (~5.8) and demonstrated that extraction techniques, such as ultrasound, may differentially affect the recovery of RuBisCO compared to other chlorophyll-binding proteins. RuBisCO is frequently cited as the dominant protein in duckweed species and is recognized as a nutritionally high-value leaf protein owing to its balanced amino acid profile and excellent digestibility [2,29].

These literature findings align well with our results, which indicate that acidic pH (pH 3) and moderate heating (85 °C) facilitate protein precipitation in DWG extracts. Given that RuBisCO and other globulin-type proteins are sensitive to denaturation, the decline in yield observed at 95 °C may be attributed to irreversible unfolding and aggregation of these major proteins under excessive thermal stress. Collectively, these results emphasize the importance of process optimization for extracting functional protein ingredients from *W. globosa*. The combined use of pH 3 and 85 °C thus represents favorable conditions for maximizing protein recovery, providing a robust foundation for large-scale production of plant-based protein isolates for food and nutraceutical applications.

### 3.6. Total Phenolic Content

The total phenolic content (TPC) of DWG protein extracts was significantly influenced by both pH and temperature during the extraction process. The highest TPC was observed at pH 5, indicating that mildly acidic conditions are favorable for preserving phenolic compounds. This outcome is consistent with recent literature showing that polyphenols remain more stable in acidic environments. In contrast, exposure to alkaline pH accelerates their oxidative degradation and facilitates polymerization or irreversible binding to proteins [30,31]. Alkaline conditions are known to disrupt phenolic stability through quinone formation and protein–phenol interactions, ultimately decreasing measurable free phenolics and overall bioactivity. Hence, protein precipitation under mildly acidic conditions appears optimal not only for protein recovery but also for retaining phenolic compounds that contribute to biological functionality. In contrast, protein precipitation at elevated temperatures, particularly at 65 °C, resulted in significantly reduced phenolic content. This decline is likely due to the thermal sensitivity of phenolic compounds, which are prone to structural breakdown and oxidation at high temperatures. Similar findings have been reported in other plant-based systems, where temperatures exceeding 80 °C led to substantial reductions in TPC and antioxidant activity as a result of polyphenol degradation [32,33]. Additionally, heat-induced protein aggregation may hinder the release or solubilization of bound phenolics, further contributing to lower yields. Therefore, carefully controlling extraction parameters, particularly by avoiding excessive heat, is critical for preserving the phenolic fraction in protein-rich extracts.

### 3.7. Antioxidant Activities

Antioxidant data, in line with the findings on protein and phenolic contents, indicate that DWG protein extracts obtained through acid precipitation demonstrated significantly higher antioxidant properties than those obtained through temperature precipitation (*p* < 0.05). The radical scavenging activity (TEAC) and ferric-reducing antioxidant power (FRAP) of the extracts were closely associated with their phenolic content, confirming that polyphenolic constituents play a significant role in antioxidant performance. In contrast to the initial assumption that elevated temperatures markedly reduce phenolic content, our statistical analysis revealed no significant differences in total phenolic content among protein precipitates recovered at temperatures ranging from 65 to 95 °C (Figure 3b). The slight variations observed are more plausibly attributed to partial oxidation or conformational modification of thermolabile phenolics, rather than a consistent decline with rising temperature. Conversely, extracts precipitated at pH 5 displayed the highest TEAC and FRAP values, consistent with their elevated phenolic content. In contrast, those obtained at lower pH values exhibited substantially lower radical-scavenging activities.

Previous studies have shown that amino acids such as cysteine, tryptophan, and tyrosine may contribute to ABTS activity [34]. However, our findings suggest that the antioxidant activity of DWG protein extracts arises primarily from phenolic compounds rather than protein fractions. This interpretation aligns with Pagliuso et al. [35], who identified a diverse range of phenolic constituents in Wolffia species. Although this study did not perform direct profiling of individual phenolic compounds, previous LC-ESI-QTOF-MS/MS and HPLC analyses of *Wolffia globosa* have identified multiple phenolic acids, such as caffeic, ferulic, gallic, and p-coumaric acids, as well as flavonoids, including apigenin, luteolin, and kaempferol derivatives [2,7,36]. These bioactive compounds are well recognized for their strong radical-scavenging and ferric-reducing activities. Their structural features, particularly the number and position of hydroxyl and methoxy groups, govern their redox potential and ability to stabilize free radicals [37]. Therefore, the antioxidant efficiency observed in the pH 5 extract is likely attributed to a higher retention of these polyphenolic compounds.

Additionally, pH and temperature have been shown to influence the antioxidant activities of plant matrices, such as sweet potato leaves, where extracts at pH 5–7 exhibited greater activity than those at pH levels below 5 or above 7 [38]. This trend is consistent with our results, suggesting that moderate acidity favors the stabilization of phenolic structures and their interaction with protein matrices, enhancing both solubility and radical-scavenging efficiency.

Overall, these findings provide valuable insight into optimizing extraction conditions to preserve phenolic integrity and maximize the functional and antioxidant potential of *W. globosa* protein extracts. The combination of moderate acidity (pH 5) and controlled heating (≤85 °C) appears optimal for maintaining phenolic stability, thereby supporting the development of health-promoting ingredients from *W. globosa* for use in functional foods and nutraceutical applications. Due to their high protein content and antioxidant properties, the DWG protein extracts from the pH 3 precipitate (P-DWG) and pH 5 supernatant (S-DWG) were selected for further investigations on cell viability and anti-inflammatory effects.

### 3.8. THP-1 Cell Cytotoxicity Assay

THP-1 cells, a widely recognized human monocytic cell line, serve as a robust In Vitro model for investigating innate immune responses and evaluating the immunomodulatory properties of bioactive compounds [39]. In this study, THP-1-derived macrophages were utilized to assess the cytotoxicity of protein extracts from DWG, following prior evidence suggesting that plant-derived compounds can modulate pro-inflammatory cytokine production in macrophage systems [40,41]. The MTT assay was performed over a wide concentration range (0–1000 µg/mL), revealing that both the supernatant (S-DWG) and precipitate (P-DWG) extracts exerted no adverse effects on cell viability. Cell survival consistently exceeded 90% across all tested concentrations, and neither IC_10_ nor IC_50_ values could be determined, confirming a complete absence of cytotoxicity.

This excellent safety profile underscores the biocompatibility of DWG protein extracts, even at high doses, and supports their suitability for subsequent mechanistic and functional assays. These findings are in agreement with prior reports, including the study by Duangjarus et al. [42], which demonstrated that peptide fractions from *W. globosa* were non-cytotoxic to RAW 264.7 macrophages up to 1200 µg/mL. Comparable results have also been reported for other plant-derived proteins. For example, enzymatically hydrolyzed proteins from Moringa oleifera seeds [14] and lentils (*Lens culinaris*) [43] exhibited minimal cytotoxicity in macrophage models. In the case of aquatic plants, Lemna minor—a closely related duckweed species—showed no cytotoxic or apoptotic effects on human peripheral blood mononuclear cells (PBMCs), indicating high tolerance by immune cells [44]. Similarly, mannose-conjugated soybean peptides demonstrated no significant toxicity across various cell lines, including macrophages, even at concentrations up to 1000 µg/mL, with only marginal effects observed at the upper dose limit [45].

Collectively, these findings emphasize the broad safety margin of plant-based protein extracts for biomedical and nutritional applications. In particular, the consistently high cell viabilities observed in DWG-treated THP-1 cells not only confirm the extracts’ non-cytotoxic nature but also reinforce their potential as functional food components or nutraceutical ingredients. As cytotoxicity assessment constitutes a critical first step in evaluating the safety of bioactive compounds, these results provide a solid foundation for the continued exploration of DWG proteins in health-oriented applications.

### 3.9. Effect of DWG Extracts on Pro-Inflammatory Cytokine Production

Protein extracts derived from DWG significantly suppressed IL-1β production in LPS-stimulated THP-1-derived macrophages, underscoring their strong immunomodulatory capacity. IL-1β is a pivotal cytokine involved in the early stages of the innate immune response, playing a central role in the development and progression of various chronic inflammatory disorders. The standard curves for IL-1β and IL-6 are shown in Figures S5 and S6 of the Supplementary Data. The lower and upper limits of quantitation (LLOQ/ULOQ) are indicated on each graph. The quantitation range for IL-1β was approximately 0.1–1100 pg/mL, and for IL-6 approximately 0.5–1000 pg/mL, within which all results were obtained.

The pronounced reduction in IL-1β levels observed following treatment with both S-DWG and P-DWG suggests that these protein fractions contain bioactive constituents capable of modulating upstream inflammatory signaling cascades or directly interfering with inflammasome-mediated cytokine maturation. This mechanistic insight is corroborated by recent literature. For instance, ethyl acetate extracts from *Moringa oleifera* leaves significantly downregulated IL-1β and IL-6 in LPS-stimulated macrophages by inhibiting the NF-κB and COX-2 pathways, with β-sitosterol and diosphenol identified as primary active compounds [42]. Likewise, lentil hull extracts have been shown to suppress IL-1β and nitric oxide production by downregulating TLR4 and promoting an anti-inflammatory cytokine profile, including enhanced IL-10 expression [46]. Similarly, soybean-derived peptides such as lunasin exert anti-inflammatory effects by attenuating NF-κB nuclear translocation and MAPK activation, resulting in reduced expression of IL-1β, IL-6, and TNF-α [47].

Moreover, emerging evidence highlights the NLRP3 inflammasome as a critical target for plant-based anti-inflammatory agents. Extracts from *Aiouea padiformis*, for example, have been shown to inhibit NLRP3 ATPase activity, thereby preventing inflammasome assembly and subsequent IL-1β release [48]. This mechanism offers a compelling explanation for the selective effect of DWG protein extracts on IL-1β production, which appeared more pronounced than their impact on IL-6. Interestingly, the response pattern of IL-6 was opposite to that of IL-1β, showing a slight increase compared with the LPS control. This divergence suggests that DWG protein extracts may selectively inhibit inflammasome-dependent IL-1β maturation without broadly suppressing NF-κB–mediated cytokine transcription. Since IL-6 is primarily regulated at the transcriptional level, this cytokine could remain unaffected—or even upregulated—as part of a compensatory feedback mechanism that supports tissue repair and resolution of inflammation [49]. Such differential modulation between IL-1β and IL-6 has also been observed for other plant-derived immunomodulators, indicating a degree of mechanistic specificity rather than nonspecific immune suppression [48].

Such a pattern suggests a degree of mechanistic specificity, potentially involving post-transcriptional regulation or inflammasome inhibition rather than broad transcriptional suppression. Additionally, *W. globosa*-derived phytosterols have been reported to downregulate nitric oxide synthesis in macrophage models without exerting cytotoxic effects, further supporting the anti-inflammatory potential of its bioactive components [50].

Regarding potential food or nutraceutical applications, the effects observed in this study should also be considered in the context of gastrointestinal digestion. Hydrolysis of plant proteins during digestion is known to generate bioactive peptides that can modulate cytokine signaling. For example, the digestion of soybean and lentil proteins has been shown to yield peptides that inhibit NF-κB activation and reduce the production of IL-1β and IL-6 [51]. Thus, it is plausible that digestion of DWG protein extracts could release small peptides that retain or enhance anti-inflammatory activity through similar mechanisms. Further studies simulating gastrointestinal conditions are warranted to confirm the stability and activity of these bioactive components.

Taken together, these findings position DWG protein extracts as a promising class of plant-derived immunomodulators that function by reducing NF-κB pathway activation. Their ability to selectively attenuate IL-1β production without impairing cell viability suggests a favorable safety and efficacy profile. As such, DWG protein extracts hold strong potential for integration into next-generation functional foods and nutraceuticals designed to mitigate low-grade, chronic inflammation and support immune homeostasis.

### 3.10. Effects of DWG Extracts on LPS-Induced NF-kB and COX-2 Pathway

Protein extracts from DWG demonstrated potent anti-inflammatory activity by inhibiting both upstream and downstream components of the NF-κB signaling cascade in LPS-stimulated THP-1 macrophages. Western Blot analysis revealed significant reductions in phosphorylated IκB-α and NF-κB p65, preventing nuclear translocation and transcriptional activation of inflammatory genes. This suppression led to a marked downregulation of COX-2 expression, a key enzyme in prostaglandin-mediated inflammation. These dual inhibitory effects align with recent findings on other plant-derived bioactives. For example, peptides from walnuts have been shown to inhibit IκB-α phosphorylation and NF-κB translocation, thereby reducing the expression of COX-2 and pro-inflammatory cytokines [52]. Similarly, rice bran and rapeseed protein hydrolysates suppressed LPS-induced COX-2 and iNOS by attenuating the NF-κB and MAPK pathways [47]. Some plant peptides even target early LPS recognition by reducing TLR4 activity or directly binding LPS, offering a broad immunomodulatory potential. Mechanistically, DWG extracts appear to act similarly, stabilizing IκB-α to keep NF-κB inactive and limiting COX-2 transcription. The selectivity and effectiveness of this action, achieved without cytotoxicity, underscore the biofunctionality of DWG proteins. Such modulation of inflammatory signaling is highly relevant in the development of functional foods, where dietary peptides are increasingly explored as safe, natural alternatives to pharmaceuticals. Taken together, the ability of DWG extracts to suppress NF-κB activation and COX-2 expression positions them as promising candidates for anti-inflammatory nutraceuticals. Their efficacy, comparable to other well-studied plant peptides, supports their potential role in managing low-grade chronic inflammation through food-based interventions.

### 3.11. Limitations and Future Work

First, these findings were generated in In Vitro models that may not recapitulate tissue-level microenvironments or pharmacokinetics. Second, we did not perform endotoxin screening (e.g., LAL assay) nor did we include a polymyxin B control; future experiments will incorporate these to exclude LPS-mediated effects. Third, we have not yet completed compositional profiling to define phenolic subclasses or peptide constituents; targeted LC–MS and peptidomics will be used to characterize bioactive components. Ultimately, In Vivo validation is necessary to assess the bioavailability, safety, and efficacy of a compound. Moreover, we will perform formal dose–response testing coupled with bioassay-guided fractionation (e.g., solvent partitioning and chromatographic sub-fractionation) to isolate and identify active moieties. We will integrate targeted phenolic LC–MS and peptidomics to link specific constituents to activity, and we will benchmark DWG against representative plant proteins to contextualize potency and mechanism.

## 4. Materials and Methods

### 4.1. Chemical Reagents

The following reagents and materials were used in this study: Folin–Ciocalteu reagent (Merck, Darmstadt, Germany), sodium carbonate (Sigma, Darmstadt, Germany), 3-ethyl-benzothiazoline-6-sulfonic acid (ABTS) (Sigma, Darmstadt, Germany), 6 hydroxy-2,5,7,8-tetramethylchromane-2-carboxylic acid (Trolox), TPTZ (2,4,6-tris [2-pyridyl]-s-triazine]), petroleum ether (J.T. Baker, Pune, India), ethanol 95% (Merck, Darmstadt, Germany), gallic acid (Sigma, Germany), amino acid standard (Sigma-Aldrich, St. Louis, MO, USA)

### 4.2. Chemical Compositions

Fresh *W. globosa* was obtained in April 2021 from a natural pond in the San Pa Tong (ST) district, Chiang Mai province, northern Thailand (18.649283° N, 99.124908° E). A voucher specimen (No. 010112) was stored in the herbarium of the Faculty of Pharmacy, Chiang Mai University (CMU), Thailand. The fresh *W. globosa* was dehydrated using a hot air oven (GmbH Company, Berlin, Germany) set at a temperature of 55 °C for 8 h until the water content of *W. globosa* was reduced to less than 10%. Dried *W. globosa* (DWG) was stored at room temperature for further study.

#### 4.2.1. Heavy Metal Analysis of DWG

Heavy metals, including arsenic (As), cadmium (Cd), mercury (Hg), and lead (Pb), were analyzed using AOAC method 2013.06 (2013) with appropriate sample digestion and quantification Via Inductively Coupled Plasma Mass Spectrometry (ICP-MS) or Graphite Furnace Atomic Absorption Spectroscopy (GFAAS), depending on the metal. Tin (Sn) was analyzed following AOAC method 985.16 (2013) using Flame Atomic Absorption Spectroscopy (FAAS). For all metals, sample digestion was performed using a closed-vessel microwave digestion system with a mixture of nitric acid and hydrogen peroxide. Quality assurance measures included the use of certified reference materials (CRMs) and blanks to validate accuracy and precision. Limits of detection (LOD) and limits of quantification (LOQ) were determined based on standard protocols.

#### 4.2.2. Proximate Analysis of DWG

The proximate analysis of DWG was analyzed using the protocols of the Association of Official Analytical Chemists (AOAC 2012) [53].

- Protein content: The protein content was measured by nitrogen analysis using a combustion method with a LECO Nitrogen and Protein Analyzer (FP828, LECO Corporation, St. Joseph, MI, USA). Approximately 0.2 ± 0.05 g of DWG and EDTA, used as a standard, were precisely weighed. The samples were carefully wrapped in tin foil to preserve their integrity and then placed into the protein analyzer. The protein content was calculated by multiplying the nitrogen content by a factor of 6.25.
- Carbohydrate content: The carbohydrate content was calculated using the formula 100—(the sum of moisture, protein, fat, and ash).
- Total fat content (method 991.36): 3 g of DWG was used to determine the overall fat content using the Soxhlet extraction method (Buchi, E-800, UAE, Büchi Labortechnik AG, Flawil, Switzerland) with petroleum ether as the solvent.
- Ash content (method 923.03): 3 g of DWG was dried at 550 °C in a combustion chamber (Vulcan, 3-550, Kitzingen, Germany). After the sample turned gray or white, the next stage involved measuring its weight to quantify the percentage of ash content.
- Moisture content (method 934.01): Approximately 2–3 g of DWG were analyzed for moisture content by drying at 105 °C until a constant weight was achieved. Afterwards, the moisture level was measured.

#### 4.2.3. Amino Acid Composition

The amino acid composition was modified from Hunsakul et al. [54]. A 50 g sample of DWG was fully hydrolyzed by treating it with a 6 N HCl solution containing 1% phenol for 24 h at 110 °C. After hydrolysis, the sample underwent derivatization with phenyl isothiocyanate for 10 min at 25 °C. The amino acid composition analysis was conducted using the HPLC system manufactured by Shimadzu in Kyoto, Japan. The system was fitted with a Shim-pack Amino-Na column (5 µm, 46.0 × 100 mm), also manufactured by Shimadzu. The RF20A detector, also from Shimadzu, was used in the analysis. The flow rate was adjusted to 0.4 mL/min and observed at a wavelength of 280 nm. The amino acid compositions were determined by utilizing an amino acid standard.

### 4.3. Protein Extraction of DWG

The protein from DWG was extracted by macerating the sample in a sodium hydroxide solution (pH 9) at DWG:solvent ratio of 1:300 (*w*/*v*), and incubating at room temperature with a mixing speed of 60 rpm for 1 h. The mixture was then ground and filtered through Whatman paper No. 4 (Cytiva, Marlborough, MA, USA). The protein was then precipitated by acid (adjusting with hydrochloric acid to achieve pH levels of 2, 3, 4, and 5) and heating (temperature 65, 75, 85, and 95 °C). The supernatant (soluble protein) and precipitate (insoluble protein) extracts from each sample were separated by centrifugation at 5000 rpm for 10 min at 4 °C and then dehydrated using a freeze-dryer (Martin Christ Control LD Plus, Martin Christ Gefriertrocknungsanlagen GmbH, Osterode am Harz, Germany). The resulting supernatant from DWG (S-DWG) and the precipitate from DWG (P-DWG) were stored at 4 °C for further analysis. The protein extraction yield of DWG was calculated using the following Equation (1):

(1) $$yield\left(\%\right)=\frac{weight\;of\;the\;extract\;from\;DWG\;powder\;(g)}{weight\;of\;DWG\;(g)}\times 100$$

### 4.4. Total Phenolic Content (TPC) Assay

The TPC assay of the S-DWG and P-DWG was determined using the Folin–Ciocalteu method described by Khonkarn et al. [55]. In this procedure, approximately 20 µL of S-DWG or P-DWG at a concentration of 2 mg/mL total extract mass was combined with 150 µL of 10% (*v*/*v*) Folin–Ciocalteu reagent. The mixture was allowed to react at ambient temperature for about 3 min. Subsequently, 100 µL of 7.5% (*w*/*v*) sodium carbonate was added, and the mixture was incubated in the dark for 30 min. The absorbance was then measured at 760 nm using a microplate reader (SPECTROstar Nano, BMGLABTECH, Ortenberg, Germany). Gallic acid was used as the standard, and the TPC results were expressed as gallic acid equivalents (GAE) (µM/mL).

### 4.5. Antioxidant Assays

#### 4.5.1. The 2,2′-Azino-bis(3-ethylbenzothiazoline-6-sulfonic acid) (ABTS) Assay

The ABTS assay for the S-DWG and P-DWG was conducted according to the method described by Khonkarn et al. [55]. A stock solution was prepared by mixing 7 mM 2,2-azinobis-3-ethyl-benzothiazoline-6-sulfonic acid (ABTS) reagent with 2.45 mM potassium persulfate (K_8_S_2_O_8_) in a 1:1 (*v*/*v*) ratio. This mixture was allowed to react at ambient temperature for 12 h. Afterward, the ABTS stock solution was diluted with deionized water until the absorbance at 734 nm reached 0.70 ± 0.05, as measured using a microplate reader. For the assay, 10 µL of S-DWG/P-DWG at a concentration of 2 mg/mL total extract mass was added to 290 µL of the ABTS stock solution and incubated in the dark for 5 min. The absorbance of the resulting mixture was measured at 734 nm using a microplate reader (SPECTROstar Nano, BMGLABTECH, Germany). Ethanol was used as the negative control, and the results were expressed in mmol Trolox/mg.

#### 4.5.2. Ferric Reducing Antioxidant Power (FRAP) Assay

The FRAP assay of the S-DWG and P-DWG was performed according to the method outlined by Khonkarn et al. [55]. The FRAP stock solution was prepared by mixing 300 mM acetate buffer, pH 3.6 (comprising 3.1 g sodium acetate trihydrate and 16 mL glacial acetic acid, diluted to 1:1 with deionized water), 10 mM TPTZ (2,4,6-tris [2-pyridyl]-s-triazine) in 40 mM HCl, and 20 mM FeCl_3_.6H_2_O in a ratio of 10:1:1 (*v*/*v*/*v*). The resulting reagent should exhibit a slightly orange color. For the assay, 15 µL of S-DWG/P-DWG (concentration of 2 mg/mL total extract mass) was added to 285 µL of the FRAP reagent and incubated at ambient temperature for 5 min. The absorbance was then recorded at 595 nm using a microplate reader (SPECTROstar Nano, BMGLABTECH, Ortenberg, Germany). A standard calibration curve of Trolox was used to estimate the antioxidant capacity of the samples, with deionized water serving as the negative control. The results were reported as mmol Fe^2+^/mg.

### 4.6. THP-1 Cell Preparation

The THP-1 human monocytic cell line (ATCC) was cultured at a density of 2 × 10^5^ cells/mL in RPMI 1640 medium supplemented with 10% fetal bovine serum and 1% antibiotic-antimycotic solution (Thermo Fisher Scientific, Waltham, MA, USA). Differentiation into macrophages was induced by treating the cells with 20 ng/mL Phorbol 12-myristate 13-acetate (PMA) (Sigma-Aldrich, St. Louis, MO, USA) and then allowing them to rest for 72 h post-PMA. The cells were maintained in 24-well plates at 37 °C with 5% CO_2_ for 72 h. The medium was refreshed daily to facilitate differentiation, as confirmed by flow cytometry.

### 4.7. Cell Cytotoxicity Assay

Cell cytotoxicity was assessed using the MTT assay [56]. Differentiated macrophages were seeded in 96-well plates at a density of 5 × 10^4^ cells/well and incubated for 24 h before treatment with extracts at concentrations ranging from 0 to 1000 µg/mL. Following overnight incubation, cells were washed with Phosphate-Buffered Saline (PBS) and stained with 0.5 mg/mL MTT (Invitrogen, Carlsbad, CA, USA) for 3 h at 37 °C and 5% CO_2_. The resulting formazan was solubilized in DMSO, and absorbance was measured at 570 nm using a microplate reader (PerkinElmer, Waltham, MA, USA). The half-maximal inhibitory concentration (IC_50_) was determined Via sigmoidal Dose–Response analysis, and the IC_10_ value was selected as the non-toxic concentration for subsequent experiments.

### 4.8. In Vitro Anti-Inflammatory Analysis

Fully differentiated human macrophages were used to assess the anti-inflammatory properties of the extracts. The THP1-derived macrophages were seeded at a density of 1 × 10^5^ cells/well in 24-well plates and incubated under standard culture conditions for 24 h. After that, THP1-derived macrophage cells were also activated by lipopolysaccharide (LPS) (cat no.93572-42-0) (Sigma-Aldrich, St. Louis, MO, USA) at a concentration of 10 ng/mL for 6 h to stimulate an acute inflammatory response. The S-DWG and P-DWG were added to the cell culture at concentrations of 25, 50, and 100 µg/mL. The cells were then incubated in the same culture conditions for 6 h. The LPS-treated cells (without any added substance) served as a negative control condition, whereas dexamethasone was used as a positive control for anti-inflammatory effects. Cells were removed by centrifugation, and the supernatants were stored at −20 °C until further testing.

#### 4.8.1. Bio-Plex Multiplex Immunoassay

The expression levels of IL-1β and IL-6 were quantified using Bio-Plex sandwich immunoassays, as per the manufacturer’s protocol (Bio-Rad Laboratories Inc., Hercules, CA, USA). This fluorescence-based bead detection method employed a standard dilution series (S1–S8), with standard curves generated in Bio-Plex Manager software version 6.2 using Brendan Scientific StatLIA 5PL fitting (Bio-Rad Laboratories Inc., Hercules, CA, USA). Briefly, 50 µL of beads were added to 96-well plates and washed twice. Cell culture supernatants from each experimental condition were then loaded and incubated for 1 h on a shaker, followed by three washes. Next, 25 µL of detection antibody was added and incubated for 30 min, followed by three washes. Afterward, streptavidin–phycoerythrin (SA-PE) was added and incubated for 10 min. All incubation steps were performed at room temperature, with washing buffer and a magnetic washer applied at each wash. Finally, assay buffer was added to resuspend the samples before analysis with the Bio-Plex 200 reader (Bio-Rad Laboratories Inc., Hercules, CA, USA), which used a red laser (635 nm) to classify bead regions and a green laser (532 nm) to quantify SA-PE fluorescence. Sample dilutions were 1:4, and all measurements were performed in triplicate.

#### 4.8.2. SDS-PAGE and Western Blot Analysis

The cell culture for each experimental condition was washed with ice-cold PBS, and total protein was extracted by incubating with RIPA lysis buffer (Thermo Fisher Scientific, New York, NY, USA) for 30 min at 4 °C. The sample was then centrifuged at 12,000 rpm for 20 min at 4 °C. The supernatant was stored at −20 °C until further testing. The Bradford assay was used to determine the total protein concentration of the extracted sample. Next, total protein samples were prepared at a concentration of 20 mg/mL, mixed with loading dye, and boiled in water for 5 min. The obtained protein samples were loaded onto a 12% SDS–polyacrylamide gel electrophoresis (PAGE), and analyzed by electrophoresis, performed against a protein ladder. The gel was then transferred onto a polyvinylidene fluoride (PVDF) membrane (Bio-Rad Laboratories, Inc., Hercules, CA, USA). The membrane containing the protein sample was blocked with 5% bovine serum albumin (BSA) (Capricorn Scientific GmbH, Hesse, Germany) for 2 h. The membrane was blotted using primary antibodies specific to phospho-NF-κB p65 with dilution 1:1000 (cat no. MA5-15160) (Thermo Fisher Scientific, New York, NY, USA), phospho-IκB-α with dilution 1:1000 (cat no. 2859) (Cell Signaling Technology, Beverly, MA, USA), and COX-2 (cat no.sc-19999) with dilution 1:1000 (Santa Cruz, CA, USA), with shaking at 4 °C overnight. The membrane was then washed with Tris-buffered saline with Tween 20 (TBST) buffer and stained with horseradish peroxidase-conjugated goat anti-mouse IgG (H + L) secondary antibody with dilution 1:10,000 (cat no. 31430) (Thermo Fisher Scientific, Waltham, MA, USA) at room temperature for 1 h. The membrane was washed four times with TBST buffer and soaked in chemiluminescent substrate for 5 min. The photographed membrane was analyzed using a ChemiDoc XRS+ Imaging System (Bio-Rad) and Image Studio Lite software version 5.1 (LI-COR Corporate, Lincoln, NE, USA).

### 4.9. Statistical Analysis

All experiments were performed in triplicate, and the results are expressed as mean ± standard deviation (SD). One-way ANOVA followed by Duncan’s multiple range test (*p* < 0.05) was conducted to determine significant differences among treatments. The analyses included both extract types (S-DWG and P-DWG) under the same experimental factor (pH or temperature), with each factor analyzed independently. Statistical analyses were performed using SPSS software (version 20, IBM Corp., Armonk, NY, USA). One-way ANOVA with Duncan’s post hoc test was also applied to evaluate differences in antioxidant activity and total phenolic content (TPC), using the same significance criterion (*p* < 0.05). Independent t-tests were used to assess the effects of DWG extracts on pro-inflammatory cytokine production and LPS-induced NF-κB and COX-2 pathways, with statistical significance set at *p* < 0.01 and *p* < 0.001.

## 5. Conclusions

This study provides compelling evidence that protein extracts from *W. globosa*, cultivated in northern Thailand, are not only rich in nutritional value but also exhibit potent health-promoting properties. The proximate and amino acid analyses confirmed its high-quality protein content, particularly lysine, leucine, and phenylalanine. Notably, the heavy metal content of the dried samples remained well within permissible limits, affirming their safety for human consumption. Extraction optimization revealed that acid precipitation at pH 3 yielded the highest protein recovery, while pH 5 significantly enhanced phenolic content and antioxidant potential, as evidenced by ABTS and FRAP assays. These antioxidant effects correlated with phenolic content, supporting the role of polyphenols in redox modulation. Crucially, both S-DWG and P-DWG demonstrated excellent biocompatibility in THP-1 macrophages, with no observed cytotoxicity up to a concentration of 1000 µg/mL. Moreover, these extracts substantially decreased LPS-induced inflammatory signaling, as indicated by reductions in phospho-NF-κB p65, phospho-IκB-α, and COX-2 protein expression. This study highlights *W. globosa* as a safe and sustainable source of plant protein with promising potential for development as a bioactive food ingredient. While the In Vitro results provide important mechanistic insights, they may not fully capture tissue-level contexts or pharmacokinetics. Limitations include the absence of endotoxin screening and comprehensive compositional profiling; these will be addressed in future work using LAL assays, polymyxin B controls, and targeted LC–MS/peptidomics to identify active phenolic and peptide constituents. Future research will focus on In Vivo validation to assess bioavailability, safety, and efficacy, alongside dose–response studies and bioassay-guided fractionation to isolate bioactive moieties. Coupled with techno-economic assessments and scale-up studies, these efforts will establish the feasibility of integrating *W. globosa* into next-generation functional foods and nutraceuticals, contributing to both human health and sustainable agri-food innovation.

## Figures and Tables

**Table 1:** Heavy metal concentrations in DWG samples (dry weight basis) compared with Thai regulatory limits (Notification No. 414, 2020).

Heavy Metal	DWG Sample (mg/kg, Dried Weight Basis)	Thai Regulatory Limit (mg/kg) *
Arsenic (As)	2.00	≤2.0
Cadmium (Cd)	0.032	≤2.0
Lead (Pb)	0.12	≤1.0
Mercury (Hg)	0.084	≤0.5

Noted: * = Thai Ministry of Public Health Notification No. 414; Food Standards for Contaminated Substances. Ministry of Public Health: Bangkok, Thailand, 2020.

**Table 2:** Amino acid composition of DWG (dry weight basis).

List of Amino Acids	Amount (mg/100 g)
Histidine *	559.33 ± 12.90
Isoleucine *	776.67 ± 16.80
Leucine *	1343.67 ± 41.14
Lysine *	3417.67 ± 33.31
Methionine *	96.00 ± 7.55
Phenylalanine *	1360.67 ± 43.82
Threonine *	146.00 ± 8.19
Tryptophan *	83.00 ± 7.55
Valine *	825.67 ± 17.56
Total essential amino acid	8608.67 ± 107.00
Alanine	1328.67 ± 30.66
Aspartic acid	625.00 ± 26.51
Cystine	427.33 ± 16.86
Glutamic acid	753.00 ± 34.51
Glycine	333.67 ± 15.50
Hydroxylysine	<20
Hydroxyproline	<20
Proline	353.00 ± 34.51
Serine	574.33 ± 14.01
Tyrosine	831.67 ± 18.61
Total non-essential amino acid	5226.67 ± 128.10

Noted: * = essential amino acid. The data are presented as the mean ± standard deviation (SD) from three replicate measurements.

## Data Availability

The authors declare that the data supporting the findings of this study are available within the article.

## Supplementary Materials

The following supporting information can be downloaded at https://www.mdpi.com/article/10.3390/molecules30204092/s1. Table S1. Statistical analysis of protein content from S-DWG and P-DWG at different pH levels using one-way ANOVA and Duncan’s multiple range test (*p* < 0.05); Table S2. Statistical analysis of protein content from S-DWG and P-DWG at different temperature levels using one-way ANOVA and Duncan’s multiple range test (*p* < 0.05); Table S3. Statistical analysis of total phenolic content (TPC) from S-DWG and P-DWG at different pH levels using one-way ANOVA and Duncan’s multiple range test (DMRT, *p* < 0.05); Table S4. Statistical analysis of total phenolic content (TPC) from S-DWG and P-DWG at different extraction temperatures using one-way ANOVA and Duncan’s multiple range test (DMRT, *p* < 0.05). Figure S1. Original blots of Beta-actin and protein ladder; Figure S2. Original blots of COX-2 and protein ladder; Figure S3. Original blots of phospho-IkB-α and protein ladder; Figure S4. Original blots of phospho-NF-KB p65 and protein ladder; Figure S5. Standard Curves of IL-1β (5PL, StatLIA); Figure S6. Standard Curves of IL-6 (5PL, StatLIA).
