# Supplementary material for: Bioactive Potential of Protein Extracts Derived from Dried Wolffia globosa on In Vitro Antioxidant Activities and Pro-Inflammatory Cytokine Production
Source: Molecules. 2025 Oct 15;30(20):4092. doi: 10.3390/molecules30204092 (PMC12566428; doi:10.3390/molecules30204092)
Supplement: Supplementary file 1 [file molecules-30-04092-s001.zip › molecules-3925189-supplementary.pdf]

Supplementary Material

Bioactive Potential of Protein Extracts Derived from Dried *Wolffia globosa* on *in Vitro* Antioxidant Activities and Pro-inflammatory Cytokine Production

Table S1. Statistical analysis of protein content from S-DWG and P-DWG at different pH levels using one-way ANOVA and Duncan's multiple range test ($p < 0.05$).

**Sample**	**pH**	Mean ± SD	**Duncan's grouping**	**ANOVA value**
S-DWG	2	12.48±0.78	d	<0.001
	3	12.07±0.28	d	
	4	19.75 ± 0.97	c	
	5	23.95 ± 0.85	c	
P-DWG	2	49.38 ± 2.87	a	<0.001
	3	51.15 ± 6.71	a	
	4	41.68 ± 1.46	b	
	5	38.97 ± 10.69	b	

Table S2. Statistical analysis of protein content from S-DWG and P-DWG at different temperature levels using one-way ANOVA and Duncan's multiple range test ($p < 0.05$).

**Sample**	**Temperature (°C)**	Mean ± SD	**Duncan's grouping**	**ANOVA value**
S-DWG	65	23.01 ± 1.51	c	<0.001
	75	25.49 ± 1.75	bc	
	85	19.86 ± 0.42	c	
	95	27.32 ± 1.47	bc	
P-DWG	65	33.07 ± 7.99	b	<0.001
	75	35.22 ± 4.07	b	
	85	44.30 ± 9.62	ab	
	95	29.95 ± 0.89	bc	

Table S3. Statistical analysis of total phenolic content (TPC) from S-DWG and P-DWG at different pH levels using one-way ANOVA and Duncan's multiple range test (DMRT, $p < 0.05$).

**Sample**	**pH**	Mean ± SD	**Duncan's grouping**	**ANOVA value**
S-DWG	2	9.90 ± 0.15	c	< 0.001
	3	13.72 ± 0.52	b	
	4	17.99 ± 2.83	a	
	5	12.81 ± 0.21	b	
P-DWG	2	11.25 ± 0.15	b	< 0.001
	3	13.85 ± 0.95	b	
	4	18.85 ± 2.40	a	
	5	19.48 ± 1.27	a	

Table S4. Statistical analysis of total phenolic content (TPC) from S-DWG and P-DWG at different extraction temperatures using one-way ANOVA and Duncan's multiple range test (DMRT, $p < 0.05$).

**Sample**	**Temperature (°C)**	Mean ± SD	**Duncan's grouping**	**ANOVA value**
S-DWG	65	10.73 ± 0.21	b	< 0.05
	75	8.72 ± 0.67	bc	
	85	8.85 ± 2.40	c	
	95	12.29 ± 0.74	a	
P-DWG	65	7.95 ± 0.12	c	< 0.05
	75	8.51 ± 0.64	bc	
	85	9.06 ± 0.42	bc	
	95	8.02 ± 0.21	c	

Original blot gel images are provided **(Figures S1-S4).** Data for “Phum (Hexane)” and “Phum (SFE)” are not included, as they represent lipid extracts unrelated to this study. Sample labels “Phum (Sup)” and “Phum (Precipitate)” were renamed “S-DWG” and “P-DWG,” respectively. No data manipulation was performed, apart from the omission of sections not presented in the manuscript. Pro-inflammatory mediator concentrations were measured using the Bio-Plex multiplex immunoassay system (Bio-Rad, Hercules, CA, USA), a fluorescence-based bead-based detection method. Standard dilution series (S1–S8) were prepared, and curves were generated in Bio-Plex Manager with StatLIA 5PL fitting. Standard curves for IL-1β and IL-6 **(Figures S5–S6)** indicate LLOQ/ULOQ ranges of ~0.1–1,100 pg/mL and ~0.5–1,000 pg/mL, respectively, within which all results were obtained.

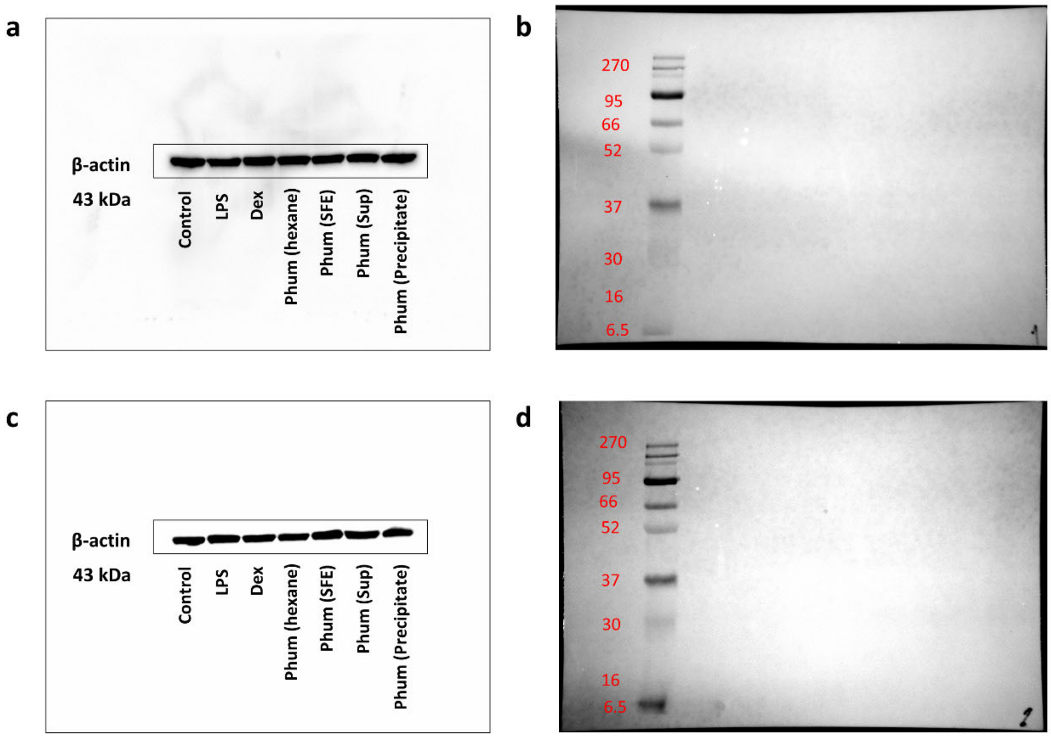

Figure S1. Original blots of Beta-actin and protein ladder.

The protein markers were obtained from Bio-Helix Co., LTD. Blultra prestained protein ladder Cat.no. PMB01-0500. These images were acquired by ChemiDoc XRS+ Imaging System (Bio-Rad Laboratories Inc., Hercules, CA, USA). (a, c) Band intensity of total protein levels of Beta-actin. It was used as loading control. (b, d) Band of Protein marker (6.5-270 kDa).

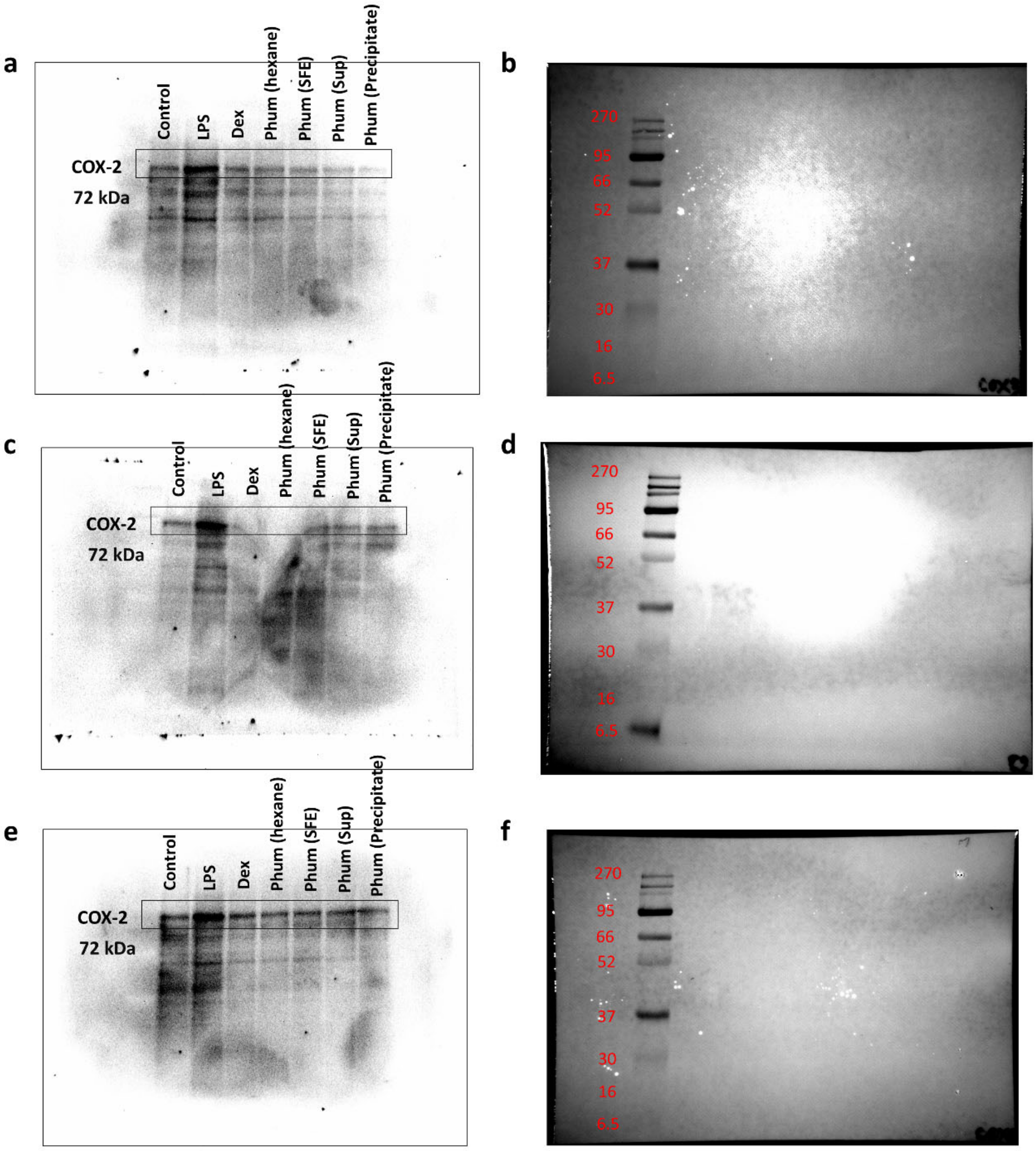

Figure S2. Original blots of COX-2 and protein ladder.

The protein markers were obtained from Bio-Helix Co., LTD. Blultra prestained protein ladder Cat.no. PMB01-0500. These images were acquired by ChemiDoc XRS+ Imaging System (Bio-Rad Laboratories Inc., Hercules, CA, USA). (a, c, e) Band intensity of total protein levels of COX-2. (b, d, f) Band of Protein marker (6.5-270 kDa).

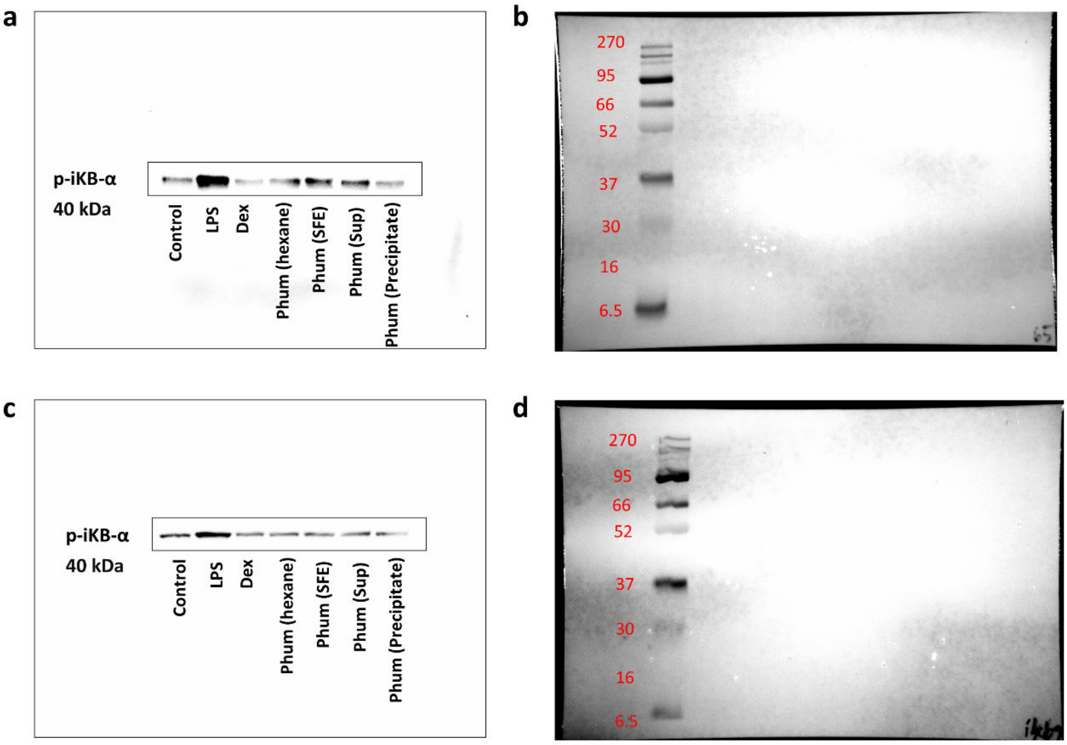

Figure S3. Original blots of phospho-IkB-α and protein ladder.

The protein markers were obtained from Bio-Helix Co., LTD. Blultra prestained protein ladder Cat.no. PMB01-0500. These images were acquired by ChemiDoc XRS+ Imaging System (Bio-Rad Laboratories Inc., Hercules, CA, USA). (a, c) Band intensity of total protein levels of phospho-IkB-α. (b, d) Band of Protein marker (6.5-270 kDa).

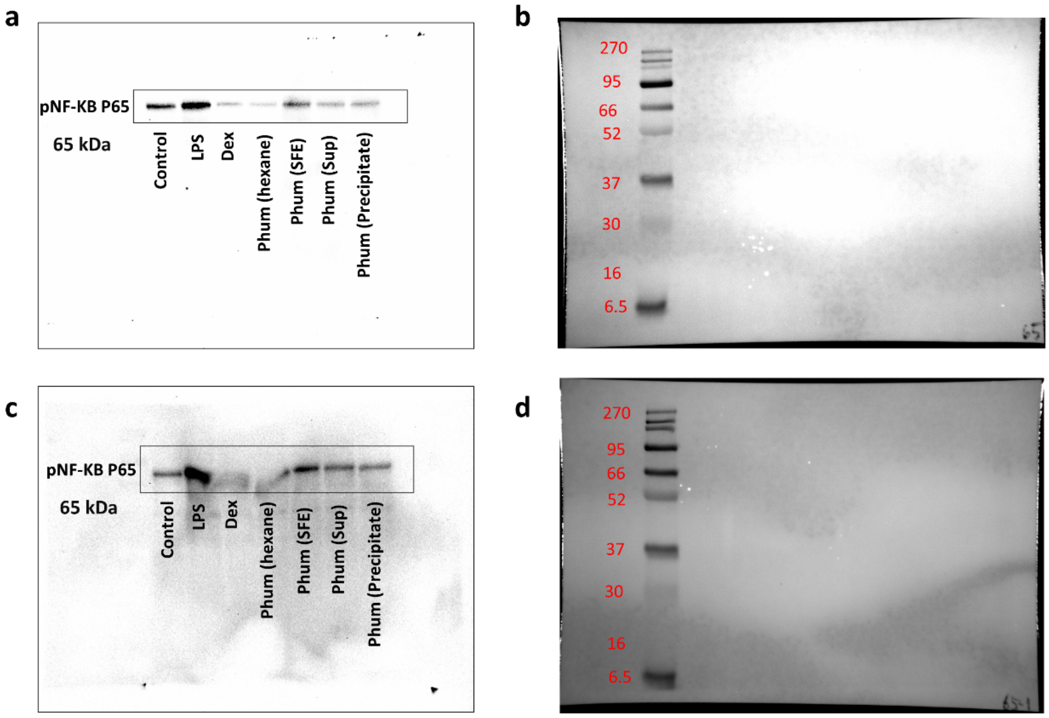

Figure S4. Original blots of phospho-NF-KB p65 and protein ladder.

The protein markers were obtained from Bio-Helix Co., LTD. Blultra prestained protein ladder Cat.no. PMB01-0500. These images were acquired by ChemiDoc XRS+ Imaging System (Bio-Rad Laboratories Inc., Hercules, CA, USA). (a, c) Band intensity of total protein levels of phospho-NF-KB p65. (b, d) Band of Protein marker (6.5-270 kDa).

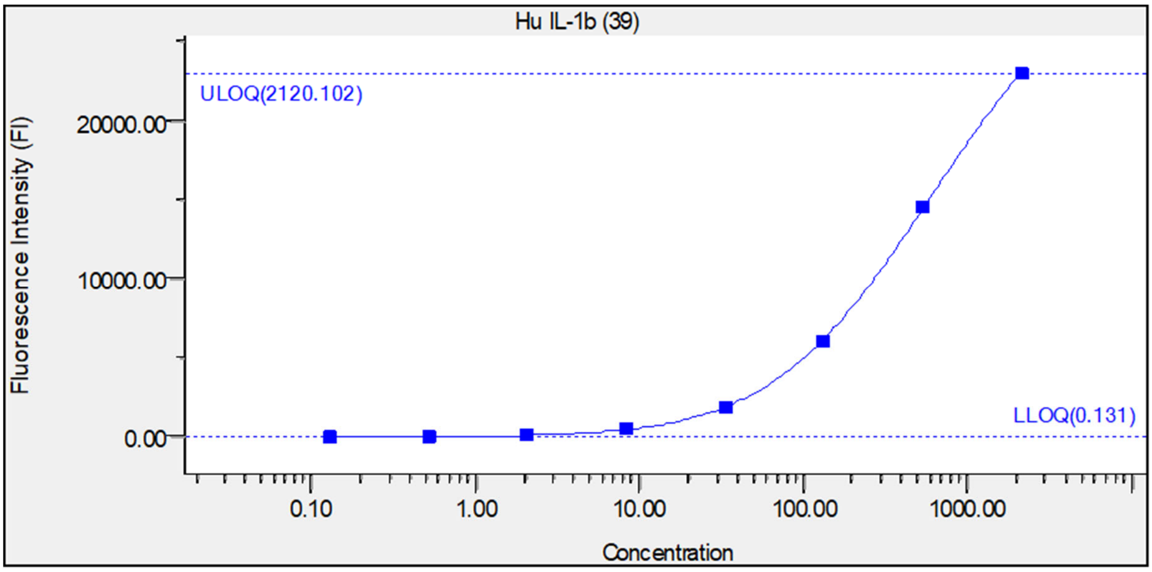

Figure S5. Standard Curves of IL-1β (5PL, StatLIA).

Regression Type: Logistic - 5PL

Std. Curve: FI = 0.797057 + (32275.6 - 0.797057) / ((1 + (Conc / 365.359)^-0.755175))^1.44166

FitProb. = 0.9601, ResVar. = 0.0998

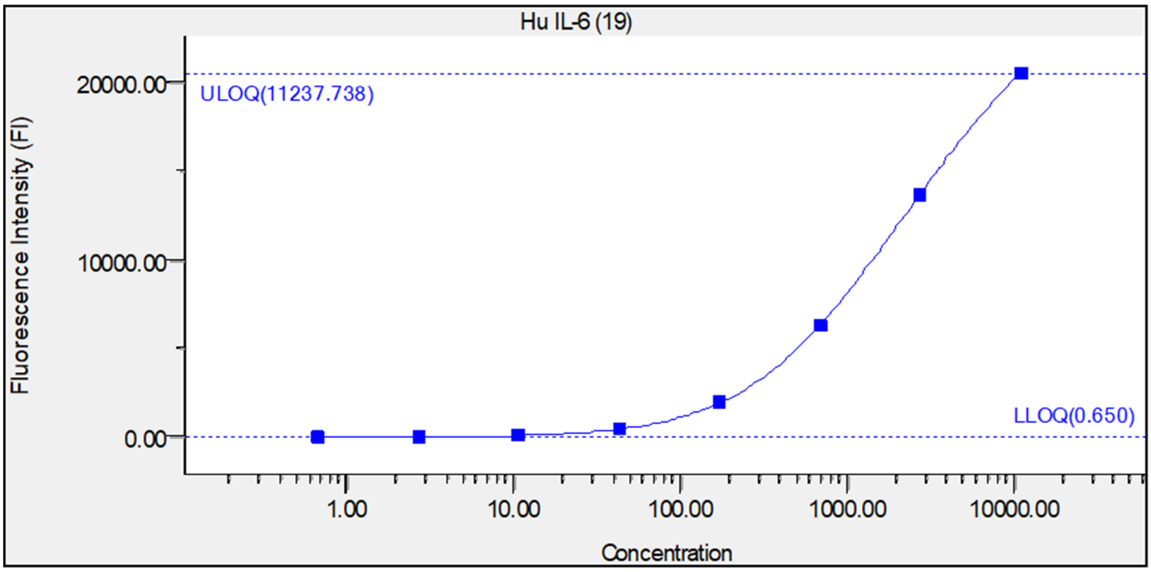

Figure S6. Standard Curves of IL-6 (5PL, StatLIA).

Regression Type: Logistic - 5PL

Std. Curve: FI = 3.22188 + (26924.7 - 3.22188) / ((1 + (Conc / 1231.73)^-0.755634))^1.55471

FitProb. = 0.9346, ResVar. = 0.1424
